# Absence of the mitochondrial translocator protein 18 kDa in mice does not affect body weight or food intake responses to altered energy availability

**DOI:** 10.1111/jne.13027

**Published:** 2021-08-22

**Authors:** Nicole A. Morrissey, Craig Beall, Kate L. J. Ellacott

**Affiliations:** ^1^ Neuroendocrine Research Group Institute of Biomedical & Clinical Sciences College of Medicine & Health University of Exeter Exeter UK

**Keywords:** high‐fat diet, metabolism, mitochondria, TSPO

## Abstract

Changes in mitochondrial function in a variety of cells/tissues are critical for orchestrating systemic energy homeostasis and are linked to the development of obesity and many of its comorbidities. The mitochondrial translocator protein of 18 kDa (TSPO) is expressed in organs throughout the body, including the brain, liver, adipose tissue, gonads and adrenal glands, where it is implicated in regulating steroidogenesis and cellular metabolism. Prior work from our group and others has shown that, in rodents, TSPO levels are altered in adipose tissue by obesity and that modulation of TSPO activity may impact systemic glucose homeostasis. Furthermore, in vitro studies in a variety of cell types have implicated TSPO in mediating cellular energetics and substrate utilisation. Although mice with germline global TSPO deficiency (TSPO^−/−^) have no reported changes in body weight under standard husbandry conditions, we hypothesised that, given the roles of TSPO in regulating mitochondrial function and cellular metabolic flexibility, these animals may have alterations in their systemic response to altered energy availability, either nutritional excess or insufficiency. In agreement with published work, compared to wild‐type (TSPO^+/+^) littermates, TSPO^−/−^ mice of both sexes did not exhibit differences in body weight on standard chow. Furthermore, following a 12‐hour overnight fast, there was no difference in weight loss or compensatory food intake during re‐feeding. Five weeks of feeding a high‐fat diet (HFD) did not reveal any impact of the absence of TSPO on body weight gain in either male or female mice. Basal blood glucose levels and glucose clearance in a glucose tolerance test were influenced by feeding a HFD diet but not by genotype. In conclusion, in the paradigms examined, germline global deletion of TSPO did not change the physiological response to deviations in systemic energy availability at the whole organism level.

## 1 INTRODUCTION

Mitochondrial function is critical for regulating both cellular and systemic energy homeostasis, with mitochondrial dysfunction within metabolic tissues being a key feature of obesity and many of its comorbidities.^1^, ^2^, ^3^ The mitochondrial translocator protein of 18 kDa (TSPO) resides in the outer mitochondrial membrane and contributes to a range of mitochondrial processes, including cellular metabolism, cholesterol transport and reactive oxygen species (ROS) production.^4^ For example, transfection of *Tspo* into Jurkat lymphocyte T‐cells, which ordinarily do not express TSPO, enhances ATP production compared to wild‐type T‐cells.^5^ Knockout of *Tspo* in Leydig MA‐10 cells, which typically express high levels of *Tspo,* enhances fatty acid oxidation (FAO).^6^, ^7^ In addition, knockdown of *Tspo* in U118MG glioblastoma cells attenuates mitochondrial ROS production in an experimental hypoxia paradigm.^8^ These studies, as well as others,^5^, ^9^, ^10^, ^11^, ^12^ suggest a key role of TSPO in various aspects of mitochondrial function including cellular energetics and substrate utilisation.

TSPO is expressed at varying levels throughout the body.^9^ It is highly enriched in metabolic tissues, including adipose tissue^13^, ^14^, ^15^ and the liver,^16^, ^17^ as well as steroidogenic organs where it is implicated in steroid hormone production^18^, ^19^; although the necessity of TSPO for steroidogenesis is debated.^20^ TSPO is also found within the brain, primarily within glia,^21^, ^22^, ^23^, ^24^ although there are also reported regional instances of neuronal localisation.^25^, ^26^ TSPO expression is altered by obesity in a tissue‐dependent manner.^13^, ^27^, ^28^ For example, previous work by our group showed reduced *Tspo* gene expression and binding of the TSPO ligand PK11195 in both white and brown adipose tissue from obese mice (both diet‐induced obese and melanocortin‐4 receptor deficient^13^). Immunohistochemical data from this study suggested that, within adipose tissue of obese animals, TSPO levels may be decreased in adipocytes but increased in adipose tissue macrophages within “crown‐like” structures,^29^ which, during obesity, are predominantly of a pro‐inflammatory phenotype.^30^ This differential pattern of TSPO expression may correlate with differences in mitochondrial function within these cell types during obesity, with adipocytes from obese animals showing mitochondrial dysfunction and decreased FAO, whereas the pro‐inflammatory adipose macrophages are in a highly glycolytic state.^31^ Taken together, these data suggest that TSPO may play a role in the pathophysiological response to obesity through modulation of mitochondrial activity, and that loss of this protein could impact the development of obesity and associated pro‐inflammatory changes leading to low‐grade tissue inflammation and an impact on systemic glucose homeostasis.^32^

When maintained on a standard chow diet, global germline TSPO knockout mice do not exhibit any differences in body weight gain.^9^ Basal blood glucose levels in these mice are also comparable to wild‐type controls. In a separate global germline, TSPO knockout line, female but not male mice had a modest but statistically significant increase in body weight, from 1 to 5 weeks of age, although data were not reported from older animals.^18^ Other global germline TSPO knockout mouse lines have also been generated and shown to be viable, although these studies did not report growth rates or body weight data.^33^, ^34^

Combined data from the in vitro metabolism studies suggests that TSPO may contribute to the regulation of cellular substrate utilisation, which is important for the physiological adaptation to changes in energy availability.^5^, ^7^ In the present study, we tested the hypothesis that global TSPO knockout mice will respond differently to deviations in energy availability than their wild‐type littermates. We utilised overnight fasting followed by re‐feeding to assess the homeostatic feeding response to energy insufficiency/negative energy balance. High‐fat feeding for 5 weeks was used to examine the response to excess energy availability/positive energy balance. We postulated that, when fed a high‐fat diet (HFD), the TSPO knockout mice would be resistant to weight gain, hepatic lipid accumulation and macrophage infiltration in adipose tissue because, in the absence of TSPO, mitochondria may be better equipped to metabolise excess free‐fatty acids as a result of enhanced FAO, resulting in reduced mitochondrial dysfunction.

## 2 MATERIALS AND METHODS

### 2.1 Animals

All animal studies were conducted in accordance with the UK Animals in Scientific Procedures Act 1986 (ASPA) and study plans were approved by the institutional Animal Welfare and Ethical Review Body at the University of Exeter.

The allele construct to knockout *Tspo* was generated at the Wellcome Trust Sanger Institute (Hinxton, UK) as part of the International Mouse Knockout Consortium. Exons 2 and 3 of the *Tspo* gene were labelled by a LacZ and a neomycin cassette flanked by LoxP sites. Following a cross with alleles expressing cre‐recombinase, a knockout of the neomycin cassette and exons 2 and 3 was made, resulting in mice with a *Tspo*‐null allele (Tspo^tm1b(EUCOMM)Wtsi^). Following the creation of founder breeders by in vitro fertilisation from sperm obtained from the European Mouse Mutant Cell Repository (https://www.eummcr.org), the cohort of mice were bred to C57BL6/N mice at MRC Harwell (Harwell, UK) to produce heterozygote offspring, which were bred in turn to produce homozygote knockout and wild‐type littermate mice, subsequently referred to as TSPO^−/−^ and TSPO^+/+^ respectively. Successful deletion was confirmed by Southern blotting using the facility at MRC Harwell.

**FIGURE 1:**
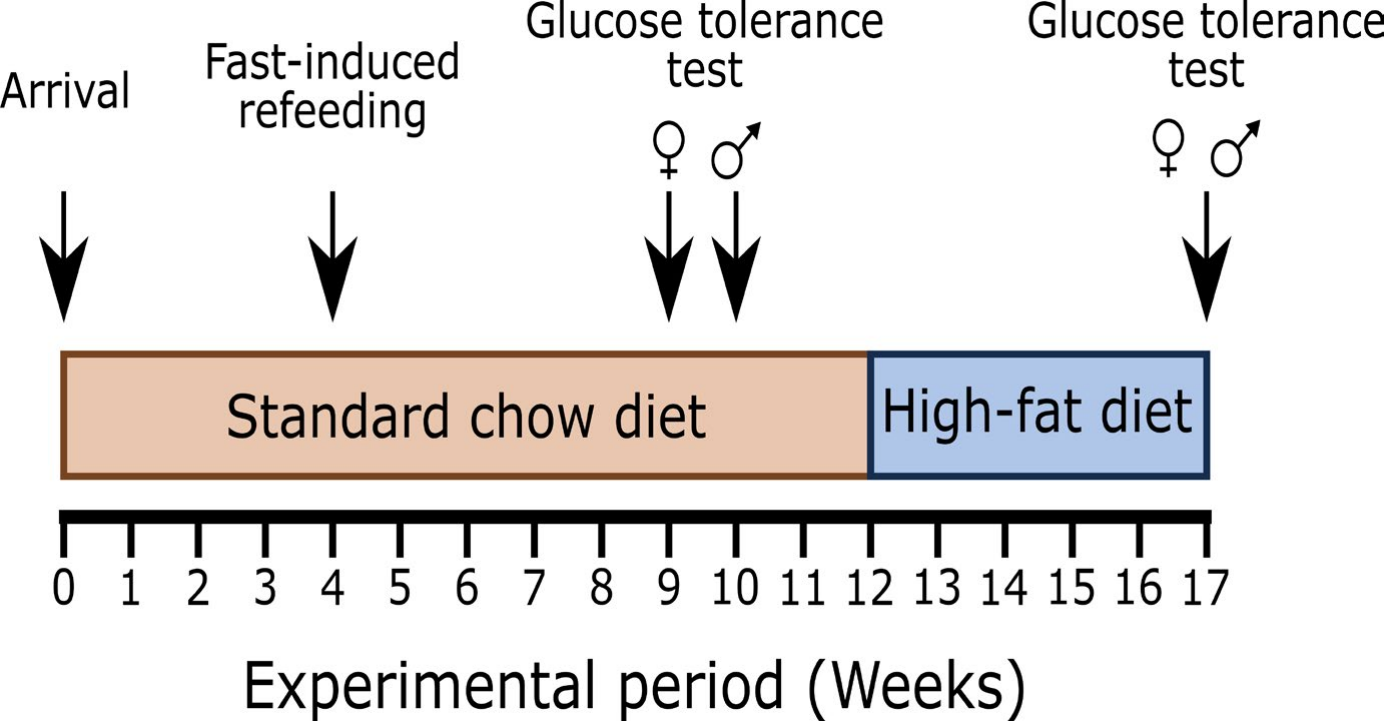
A schematic timeline of the experimental design. Individually housed mice had food intake and body weight measured weekly from experimental week 0. After 4 weeks of monitoring, all mice underwent a fast‐induced re‐feeding procedure. At experimental week 9, a subset of female mice underwent a glucose tolerance test, whereas a subset of male mice experienced the glucose tolerance test at experimental week 10. At experimental week 12 week, for all animals, the standard chow diet was replaced by high‐fat diet and weekly food intake and body weight measurements continued. After 5 weeks of a high‐fat diet, mice underwent an additional glucose tolerance test and were killed the following week

The TSPO^−/−^ mice, and their wild‐type littermate controls of both sexes, arrived at the University of Exeter as a single cohort at aged 6‐9 weeks old (Figure 1). Upon arrival, the mice were de‐identified and randomised to a unique ID by animal care technicians before being individually housed. The investigators were blinded to the genetic identity of the mice throughout the study. All animals were housed under a 12:12 hour light/dark photocycle (lights on 7.00 am) at 22 ± 2°C and 55% ± 2% humidity. Unless stated otherwise, animals had ad libitum access to standard laboratory chow (LabDiet [EU] Rodent diet 5LF2; LabDiet, St Louis, MO, USA) and water. Weekly measurements of standard chow intake and body weight were collected on Monday afternoons for 12 weeks (Figure 1). To minimise individual housing time, all animals were studied simultaneously with the age of the mice ranging between 7 and 10 weeks at week 1 of study.

In total, 12 male TSPO^+/+^, 14 male TSPO^−/−^, 13 female TSPO^+/+^ and 14 female TSPO^−/−^ mice were studied. This does not include three animals that started the study but were excluded from the statistical analysis and results reporting: one animal was revealed as heterozygous following the confirmatory genotype analysis, and two wild‐type animals were killed before the study was complete as a result of welfare concerns associated with failure to thrive/runting. The investigators were blinded to the genotype of the animals until all measurements, including image analysis, were complete.

### 2.2 Genotyping

The genetic identity of the mice was confirmed by polymerase chain reaction testing on liver samples taken from the mice following death at the end of the study. To detect the wild‐type *Tspo* allele, a 5′ arm primer sequence of AGCAGAAGTAGGAAGAAGGTG and a 3′ arm sequence of GTCAACCCATCACTGCCTTCA were used. To detect the truncated mutant *Tspo* allele present in the knockout mice, a LAR3 primer sequence of CAACGGGTTCTTCTGTTAGTCC was used in addition to the 3′ arm primer.

### 2.3 Fast‐induced re‐feeding

After 5 weeks of acclimatisation and monitoring of food intake and body weight (Figure 1), the homeostatic response of the mice to food deprivation was tested. This procedure was performed in the holding room in which the mice were kept. Within 30 minutes prior to lights out (7.00 pm), standard chow was removed from the cages and the body weight of each animal was recorded. After 12 hours, from lights on (07.00 am), standard chow was weighed and returned to the respective cages. Chow was weighed again at 1, 2, 3, 4, 12 and 24 hours following re‐introduction to assess food consumption. Body weights of the mice were taken at 0 (pre‐fasting), 12 (fasted; prior to reintroduction of food) and at 24 hours after the reintroduction of food (re‐fed).

### 2.4 Diet‐induced obesity

Twelve weeks into the study, standard chow was then replaced with HFD (TestDiet DIO 58Y1; TestDiet, St Louis, MO, USA; 60% energy from fat) as the only source of food, and weekly measurements of body weight were continued with measurements of HFD intake made three times a week (Monday, Wednesday and Friday afternoons) (Figure 1). The cage floors were checked for residual food pellets/crumbs before weighing, as some of the mice, particularly females, would hoard the HFD. As a result of limitations on ethical approval, animals were only able to be maintained on the HFD for 5 weeks (Figure 1).

### 2.5 Intraperitoneal glucose tolerance test

At lights on (7.00 am), eight male TSPO^+/+^, eight male TSPO^−/−^, nine female TSPO^+/+^ and eight female TSPO^−/−^ mice were moved to a procedure room and their food removed. To ensure representative sampling, animals of each genotype were randomly selected for assessment by an unblinded independent investigator. After 6 hours, the mice received a topical anaesthetic (EMLA cream; 2.5% lidocaine, 2.5% prilocaine; AstraZeneca, Cambridge, UK) to their tail and a small cut was made at the tip, from which blood glucose was measured by a handheld blood glucose monitor and testing strips (Codefree™; SD Biosensor, Suwon‐si, South Korea). The mice then received an i.p. injection of 2 g kg^‐1^ glucose (G7021; Sigma‐Aldrich, St Louis, MO, USA) dissolved in sterile saline (0.9% sodium chloride, S7653; Sigma‐Aldrich; injection volume 100 μL). Following glucose administration, blood glucose was measured from the tail tip cut at 15, 60, 90 and 120 minutes. This test was performed twice on the same mice during the study: once at 9‐10 weeks (prior to the introduction of the HFD) and again after 5 weeks of a HFD (Figure 1). Only a subset of the total cohort of animals were used for glucose tolerance test studies because, after completing initial studies, there was no observable effect, and so we could not support exposing animals to an unnecessary procedure that was not supported by the data.

### 2.6 Tissue collection

After 5 weeks of a HFD (Figure 1), the mice were killed by an overdose of pentobarbitone sodium (1.6 g kg^‐1^; 100 μL i.p.) followed by decapitation. Whole liver and peri‐gonadal white adipose tissue (WAT) from all mice were dissected, washed briefly in 0.01 mol L^‐1^ phosphate‐buffered saline (PBS) (RES20908‐A702X, RDD007; Sigma‐Aldrich) and weighed. A portion of liver and WAT were fixed by immersion in paraformaldehyde dissolved in PBS. Liver tissue was fixed in 4% paraformaldehyde, whereas WAT was fixed in 3% paraformaldehyde as stored at 4°C until histological processing.

### 2.7 Histological processing of liver tissue

A random subset of liver samples (six male TSPO^+/+^, seven male TSPO^−/−^, seven female TSPO^+/+^, six female TSPO^−/−^) were paraffin embedded by the Histopathology laboratory at the Royal Devon & Exeter Hospital (Exeter, UK). The paraffin‐embedded blocks were sliced using a rotary microtome into sections that were 5 μm in width and floated from a heat bath to Superfrost™ (Thermo Fisher Scientific, Waltham, MA, USA) charged glass microscope slides. The slides were left overnight to air‐dry, then deparaffinised in two changes of xylene for 10 minutes each (534056; Sigma‐Aldrich). This was followed by re‐hydration in two changes of 100% ethanol (E/0650DF/17; Thermo Fisher Scientific) for 5 minutes each, then in 95% ethanol for 2 minutes and 70% ethanol for 2 minutes. After a brief wash in distilled water, slides were incubated in haematoxylin solution (GHS316; Sigma‐Aldrich) for 90 seconds. The residual haematoxylin was washed off in warm running water for 10 minutes, followed by rinsing in distilled water. The slides were briefly dipped into 95% ethanol 10 times, and then counter‐stained in Eosin Y solution (102439; Sigma‐Aldrich) for 30 seconds. Slides were dehydrated via 5‐minute incubation in fresh 95% ethanol, followed by two 5‐minute incubations in 100% ethanol. The slides were cleared in 2 changes of xylene for 5 minutes each, and then coverslips applied with DPX mounting medium (06522; Sigma‐Aldrich). The mounting medium was left to dry, and the slides were then imaged using light microscopy (DFC340 FX; Leica, Wetzlar, Germany).

### 2.8 White adipose tissue (WAT) immunohistochemistry

Fixed WAT tissue was cut into small pieces of equal size using a scalpel and then washed in three changes of PBS for 10 minutes each. Tissue samples were incubated in 20% normal donkey serum (NDS) (D9663; Sigma‐Aldrich), diluted in PBS with 0.1% Triton X‐100 (PBS‐T) (BP151‐00; Thermo Fisher Scientific) for 1 hour at room temperature on an orbital shaker. This was followed by incubation in rat anti‐F4/80 (ab6640; Abcam, Cambridge, MA, USA) (diluted 1:250 in PBS‐T with 20% NDS) overnight at 4°C on an orbital shaker. The next morning, tissue pieces were washed three times for 5 minutes each in PBS and then incubated in donkey anti‐rat^alexa‐594^ (A‐21209; Invitrogen, Carlsbad, CA, USA) (diluted 1:1000 in PBS‐T containing 20% NDS) for 1 hour at room temperature. The tissue was washed in PBS for 10 min before incubation in rabbit anti‐TSPO (ab109497; Abcam) (diluted 1:250 in PBS‐T containing 20% NDS) for 1 hour at room temperature. This was followed by three washes in PBS for 5 minutes each, and an incubation in donkey anti‐rabbit^alexa‐488^ (ROCK611‐132‐122; VWR International, Radnor, PA, USA) (diluted 1:1000 in PBS‐T containing 20% NDS) for 1 hour at room temperature. The tissue was washed for a further 10 minutes in PBS before incubation in 1:1000 4′,6‐diamidino‐2‐phenylindole (D1306; Thermo Fisher Scientific) diluted in PBS. Tissue was mounted onto glass slides, excess liquid was dried by wicking away with tissue and coverslips were applied with fluorescent mounting medium. The sections were imaged using confocal microscopy (DMi8 TCS SP8; Leica).

### 2.9 Image analysis

Three random images per liver sample were captured using standard light microscopy. Using FIJI ImageJ, version 1.52i (National Institutes of Health, Bethesda, MD, USA), images were converted to 8‐bit inverted black‐and‐white representations of the tissue structure and the ‘Analyse particles’ feature of ImageJ was applied. This provided information including the mean area of black particles, which corresponded to the size of vacuolarised sites in the liver tissue. The mean for each of the three images taken per animal and plotted accordingly.

WAT structure was imaged at 20× magnification using a confocal microscope and 6 Z‐stacks were collated per section, one section per animal. The area of each adipocyte and number of F4/80‐postive macrophages was measured in one random WAT image per animal using ImageJ and plotted accordingly.

### 2.10 Statistical analysis

Data were processed using Excel 2013 (Microsoft Corp., Redmond, WA, USA). Graphs were produced and statistical analysis was performed using Prism, version 9 (GraphPad Software Inc., San Diego, CA, USA). Data are presented as the individual points per animal, where possible, with error bars indicating the SEM. Data were tested for normal distribution and parametric or non‐parametric statistical tests were applied, as appropriate. *P* < 0.05 was considered statistically signiifcant. For HFD intake in female mice, as a result of food shredding/hoarding in some animals, ROUT testing (ie, combining robust regression and outlier removal) was used for statistical identification of outliers.

## 3 RESULTS

### 3.1 Germline global deletion of TSPO did not affect growth curves or food intake of the mice maintained on standard diet in the absence of a homeostatic challenge

**FIGURE 2:**
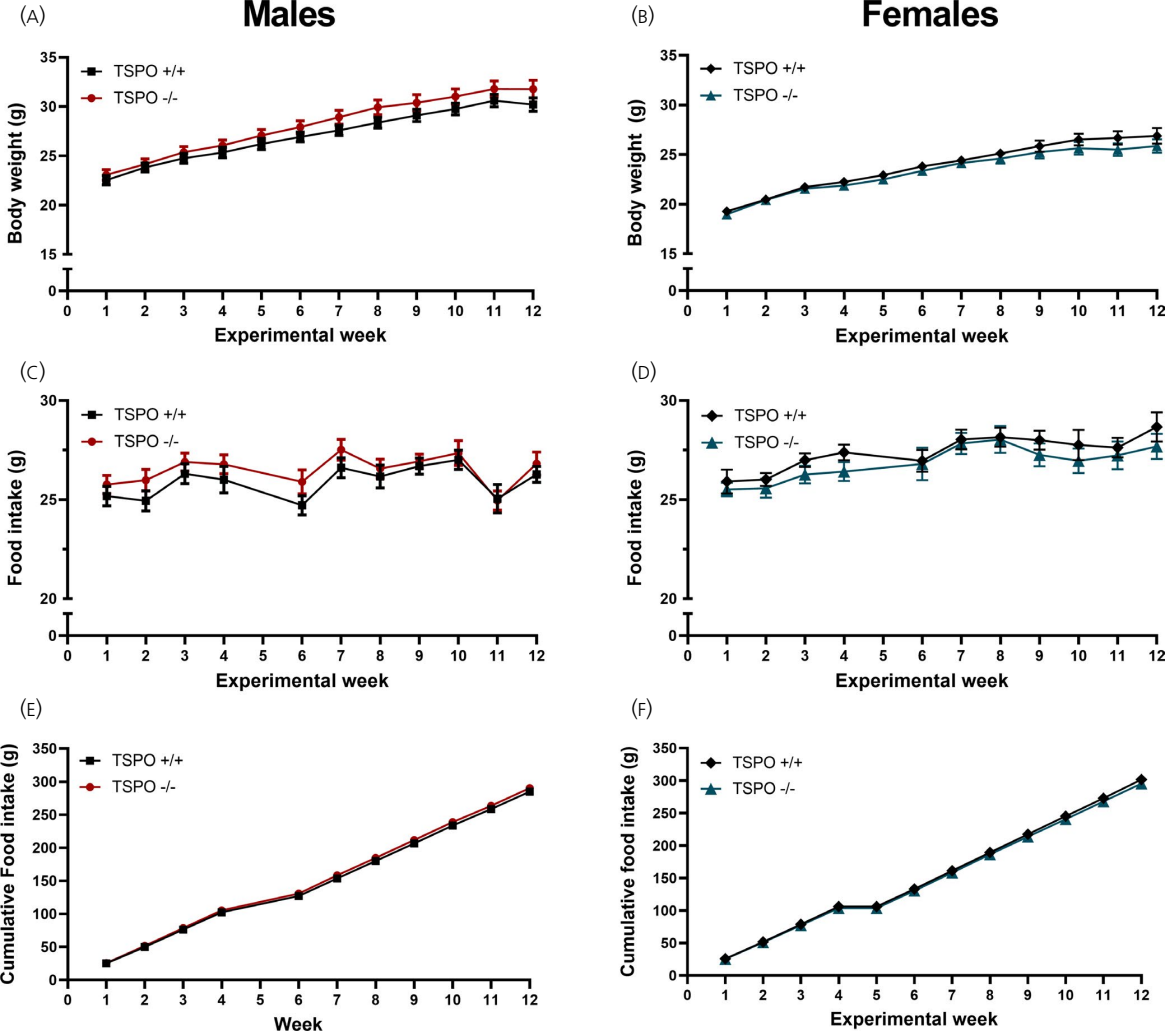
There was no difference in body weight gain or food intake between TSPO^+/+^ and TSPO^−/−^ mice maintained on standard diet. Weekly body weight gain (A, males; B, females), weekly standard chow diet food intake (C, males; D, females) and cumulative standard chow diet food intake (E, males; F, females) in TSPO^+/+^ and TSPO^−/−^ mice. Statistical analysis was performed using repeated measures two‐way ANOVA (D) or repeated measures mixed‐effects model (A‐C, E, F). No statistically significant effects of genotype were observed. Food and calorie intake data are missing from week 5 as a result of the fast‐induced re‐feeding study being performed. Data are expressed as the mean ± SE, *n* = 12‐14 mice per group. Where not seen, the error bars are too small to be visualised (E and F)

As anticipated, based on a previous report using an independently generated mouse line,^9^ there was no difference in growth curves between the TSPO^+/+^ and TSPO^−/−^ genotypes in both male (*F*_genotype 1,24_ = 1.354, *P* = 0.256, repeated measures mixed‐effect model) (Figure 2A) and female mice (*F*_genotype_ = _1,25_ = 0.854, *P* = 0.364, repeated measures mixed‐effect model) (Figure 2B) maintained on standard chow. As expected, in both sexes, there was an influence of experimental period on growth (*F*_time_ _1.9,45.7_ = 399.1, *P* < 0.0001 [males] [Figure 2A], repeated measures mixed‐effect model; *F*_time_ _2,50_ = 173, *P* < 0.0001 [females] [Figure 2B], repeated measures two‐way ANOVA). In the male cohort in the growth curves, there was a statistically significant interaction between genotype and experimental period (*F*_interaction 11,263_ = 1.93, *P* = 0.036, repeated measures mixed‐effect model) (Figure 2A), although Sidak's multiple comparisons test did not identify any statistically significant differences at any individual time point. There was no interaction between genotype and experimental period in the growth curves of female mouse cohort (*F*_interaction_ _11,275_ = 0.912, *P* = 0.529, repeated measures two‐way ANOVA) (Figure 2B).

There were no effects of genotype on weekly food intake in the male mice (*F*_genotype 1,24_ = 1.066, *P* = 0.312, repeated measures mixed‐effect model) (Figure 2C), nor in the female cohort (*F*_genotype 1,25_ = 0.762, *P* = 0.391, repeated measures mixed‐effect model) (Figure 2D). In both sexes, there was a statistically significant influence of experimental time on intake (*F*_time_ _5.5,131.4_ = 10.01, *P* < 0.0001 [males] [Figure 2C]; *F*_time_ _5.6,139.7_ = 10.55, *P* < 0.0001 [females] [Figure 2D], both analysed using repeated measures mixed‐effect model) but no interaction between time and genotype (*F*_interaction 10,239_ = 0.675, *P* = 0.747 [males] [Figure 2C]; *F*_interaction 10,249_ = 0.370, *P* = 0.959 [females] [Figure 2D], both analysed using repeated measures mixed‐effect model). Similarly, there was an effect of time but not genotype on cumulative food intake in the male (*F*_time_ _1.099,25.60_ = 7198, *P* < 0.0001; *F*_genotype 1,24_ = 1.15, *P* = 0.29, repeated measures mixed‐effect model) (Figure 2E) and female mice (*F*_time_ _1.036,25.71_ = 6214, *P* < 0.0001; *F*_genotype 1,25_ = 0.817, *P* = 0.37, repeated measures mixed‐effect model) (Figure 2F). There was no interaction between time and genotype on cumulative food intake in either sex (*F*_interaction 10,233_ = 0.748, *P* = 0.679 [males] [Figure 2E]; *F*_interaction 11,273_ = 0.546, *P* = 0.871 [females] [Figure 2F], both analysed using repeated measures mixed‐effect model).

### 3.2 Germline global deletion of TSPO did not change the homeostatic responses of mice to fasting

At week 4 of the study (Figure 1), when the mice were aged between 10 and 12 weeks, the physiological response of the animals to negative energy balance was examined. We conducted a fast‐induced re‐feeding experiment in TSPO^+/+^ and TSPO^−/−^ mice, which produced two measurements: day‐time compensatory food intake during the 24‐hour re‐feeding period following a 12‐hour overnight fast, and the body weight changes in response to fasting and re‐feeding. These measures reflect the physiological response to food deprivatresuion, and homeostatic compensation during re‐feeding: the ability of the animals to sense and recover body mass after a state of negative energy balance.

**FIGURE 3:**
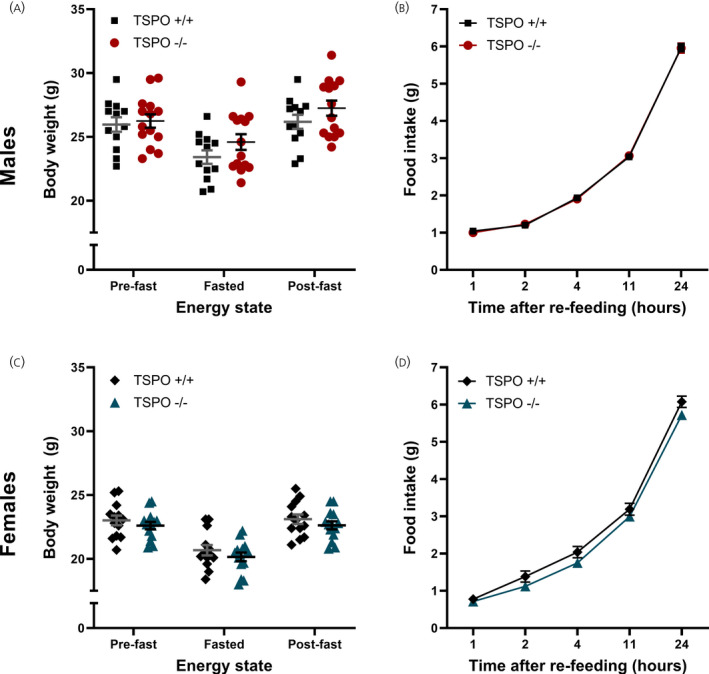
Germline global deletion TSPO had no impact on the homeostatic response to fasting and re‐feeding in either male or female mice. Mice had food removed at 7.00 pm and were fasted overnight for 12 hours before having their standard chow diet returned the following morning (7.00 am). Body weight was measured pre‐fast, after fasting (fasted) and 24 hour after the re‐introduction of food (post‐fast) (A, males; C, females). Food intake was measured at regular intervals after reintroduction at 7.00 am (B, males; D, females). No statistically significant effects of genotype were observed. Statistical analysis was performed using repeated measures two‐way ANOVA. Data are expressed as the mean ± SE, *n* = 12‐14 mice per group

In male mice, as expected, there was a significant effect of energy state on body weight (*F*_energy state_ _1.05,25.29_= 52.44, *P* < 0.0001, repeated measures two‐way ANOVA) (Figure 3A). No effect of genotype on body weight before and after food deprivation or after re‐feeding was observed (*F*_genotype 1,24_ = 1.28, *P* = 0.269, repeated measures two‐way ANOVA) (Figure 3A), nor was there an interaction between energy state and genotype (*F*_interaction 2,48_ = 1.56, *P* = 0.22, repeated measures two‐way ANOVA) (Figure 3A). As expected, there was a statistically significant effect of time on food intake during the fast‐induced re‐feeding phase in male mice (*F*_time_ _1.99,47.63_ = 2232, *P* < 0.001, repeated measures two‐way ANOVA) (Figure 3B), although no impact of genotype was observed (*F*_genotype 1,24_ = 0.002, *P* = 0.963, repeated measures two‐way ANOVA) (Figure 3B), nor was there an interaction between time and genotype (*F*_interaction 4,96_ = 0.14, *P* = 0.965, repeated measures two‐way ANOVA) (Figure 3B). Similarly, in female mice, there was a statistically significant effect of energy state on body weight (*F*_energy state_ _1.8,44.93_ = 609.2, *P* < 0.0001, repeated measures two‐way ANOVA) (Figure 3C), although there was no effect of genotype (*F*_genotype 1,25_ = 0.93, *P* = 0.345, repeated measures two‐way ANOVA) (Figure 3C) and no interaction between energy state and genotype (*F*_interaction 2,50_ = 0.25, *P* = 0.781, repeated measures two‐way ANOVA) (Figure 3C). Again, in female mice, there was a statistically significant effect of time on cumulative compensatory food intake during re‐feeding (*F*_time_ _2.29,57.14_ = 1686, *P* < 0.0001, repeated measures two‐way ANOVA) (Figure 3D), although there was no statistically significant effect of genotype on food intake during re‐feeding (*F*_genotype 1,25_ = 3.05, *P* = 0.093, repeated measures two‐way ANOVA) (Figure 3D) and no interaction between time and genotype (*F*_interaction 4,100_ = 1.23, *P* = 0.304, repeated measures two‐way ANOVA) (Figure 3D).

### 3.3 Germline global knockout of TSPO provided no protection against diet‐induced weight‐gain after 5 weeks of a HFD

**FIGURE 4:**
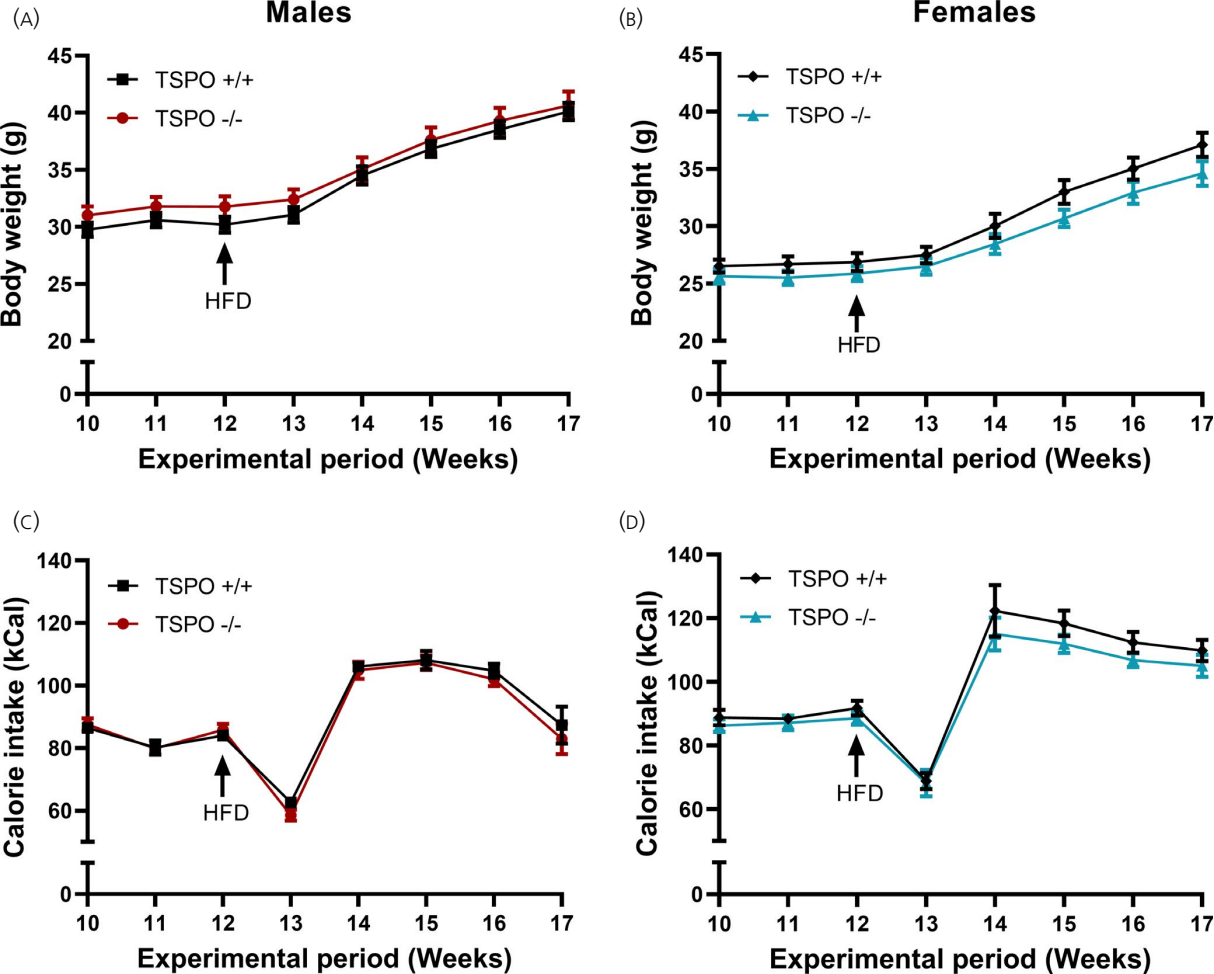
Germline global deletion of TSPO did not protect against high‐fat diet‐induced weight gain in either male or female mice. Weekly body weight (A, male; B, female) and calorie intake (C, male; D, female) of TSPO^+/+^ and TSPO^−/−^ mice fed a high‐fat diet (HFD). No statistically significant effects of genotype were observed. Statistical analysis was performed using two‐way ANOVA with repeated measures (A‐C) or a repeated measures mixed‐effects analysis (D). Data are expressed as the mean ± SE, *n* = 12‐14 mice per group. Arrow indicates the time at which standard chow diet was replaced with a HFD

Absence of TSPO had no effect on high‐fat diet‐induced body weight gain in male (*F*_genotype 1,24_ = 0.36, *P* = 0.55, repeated measures two‐way ANOVA) (Figure 4A) or female mice (*F*_genotype 1,25_ = 2.44, *P* = 0.131, repeated measures two‐way ANOVA) (Figure 4B). As anticipated, experimental time period had a significant influence on weight gain in both sexes (*F*_time_ _1.95,46.7_ = 291.4, *P* < 0.0001 [males] [Figure 4A]; *F*_time_ _2.61,65.19_ = 166.5, *P* < 0.0001 [females] [Figure 4B], both analysed using repeated measures two‐way ANOVA), although there was no interaction of genotype and experimental time period (*F*_interaction 5,96_ = 0.688, *P* = 0.6023 [males] [Figure 4A]; *F*_interaction 4,100_ = 1.23, *P* = 0.3013 [females] [Figure 4B], both analysed using repeated measures two‐way ANOVA). In both sexes, genotype alone did not have a significant influence over food intake (*F*_genotype 1,24_ = 0.988, *P* = 0.330 [males] [Figure 4C], repeated measures two‐way ANOVA; *F*_genotype 1,25_ = 2.02, *P* = 0.167 [females] [Figure 4D], repeated measures mixed‐effects analysis), whereas the experimental time period did (*F*_time_ _1.75,41.97_ = 99.31, *P* < 0.0001 [males] [Figure 4C], repeated measures two‐way ANOVA; *F*_time_ _2.78,62.64_ = 61.80, *P* < 0.0001 [females] [Figure 4D], repeated measures mixed‐effects analysis), although there was no interaction of genotype and experimental time period (*F*_interaction 4,96_ = 0.167, *P* = 0.957 [males] [Figure 4C], repeated measures two‐way ANOVA; *F*_interaction 7,158_ = 0.368, *P* = 0.92 [females] [Figure 4D], repeated measures mixed‐effects analysis). The calorie intake in the female mice was more variable than that of the males due to hoarding/shredding of the HFD pellets in some animals. To remove any potential bias, a statistical test for outliers was used resulting in nine data points (from the first or second week after the new HFD was introduced) being removed from the analysis. This was independent of genotype because there were five data points removed from the TSPO^−/−^ group and four from TSPO^+/+^ group.

In the analysis of the body weight data, when considering the relationships of all three independent variables (diet, sex, genotype) together using a three‐way ANOVA, there were statistically significant effects of diet alone and sex alone (comparing body weight at experimental week 12 [standard diet] and experimental week 17 [after 5 weeks of a HFD]). There was also a statistically significant interaction between sex and genotype, but not all three variables: sex, diet and genotype. These analyses are summarised in Table 1.

**TABLE 1:** Summary of the results of statistical analyses (three‐way ANOVA) of the relationships between genotype, sex and diet on body weight, basal blood glucose and area under the curve (AUC) of glucose clearance in the glucose tolerance test

	Body weight (g)	Basal blood glucose (mmol L^‐1^)	AUC
Diet	***F*_ 1,97_ = 205.7, *P* < 0.0001**	***F*_ 1,51_ = 27.79, *P* < 0.0001**	***F*_ 1,51_ = 65.71, *P* < 0.0001**
Sex	***F*_ 1,97_ = 48.22, *P* < 0.0001**	***F*_ 1,51_ = 4.88, *P* = 0.032**	***F*_ 1,51_ = 13.28, *P* = 0.0006**
Genotype	*F*_ 1,97_ = 0.29, *P* = 0.59	*F*_ 1,51_ = 0.40, *P* = 0.53	*F*_ 1,51_ = 0.024, *P* = 0.88
Diet × Sex	*F*_ 1,97_ = 0.29, *P* = 0.59	***F*_ 1,51_ = 5.11, *P* = 0.028**	*F*_ 1,51_ = 0.051, *P* = 0.83
Diet × Genotype	*F*_ 1,97_ = 0.93, *P* = 0.34	*F*_ 1,51_ = 0.40, *P* = 0.53	*F*_ 1,51_ = 0.11, *P* = 0.75
Sex × Genotype	***F*_ 1,97_ = 4.52, *P* = 0.036**	*F*_ 1,51_ = 1.91, *P* = 0.17	*F*_ 1,51_ = 1.29, *P* = 0.26
Diet × Sex × Genotype	*F*_ 1,97_ = 0.026, *P* = 0.87	*F*_ 1,51_ = 3.55, *P* = 0.065	*F*_ 1,51_ = 1.12, *P* = 0.30

Note

Statistically significant effects are indicated in bold.

For the body weight comparisons, the weights at experimental week 12 (standard chow diet) and experimental week 17 (after 5 weeks of a high‐fat diet) were used.

### 3.4 There was no effect of germline global deletion of TSPO on glucose tolerance in either mice fed standard chow or a HFD

**FIGURE 5:**
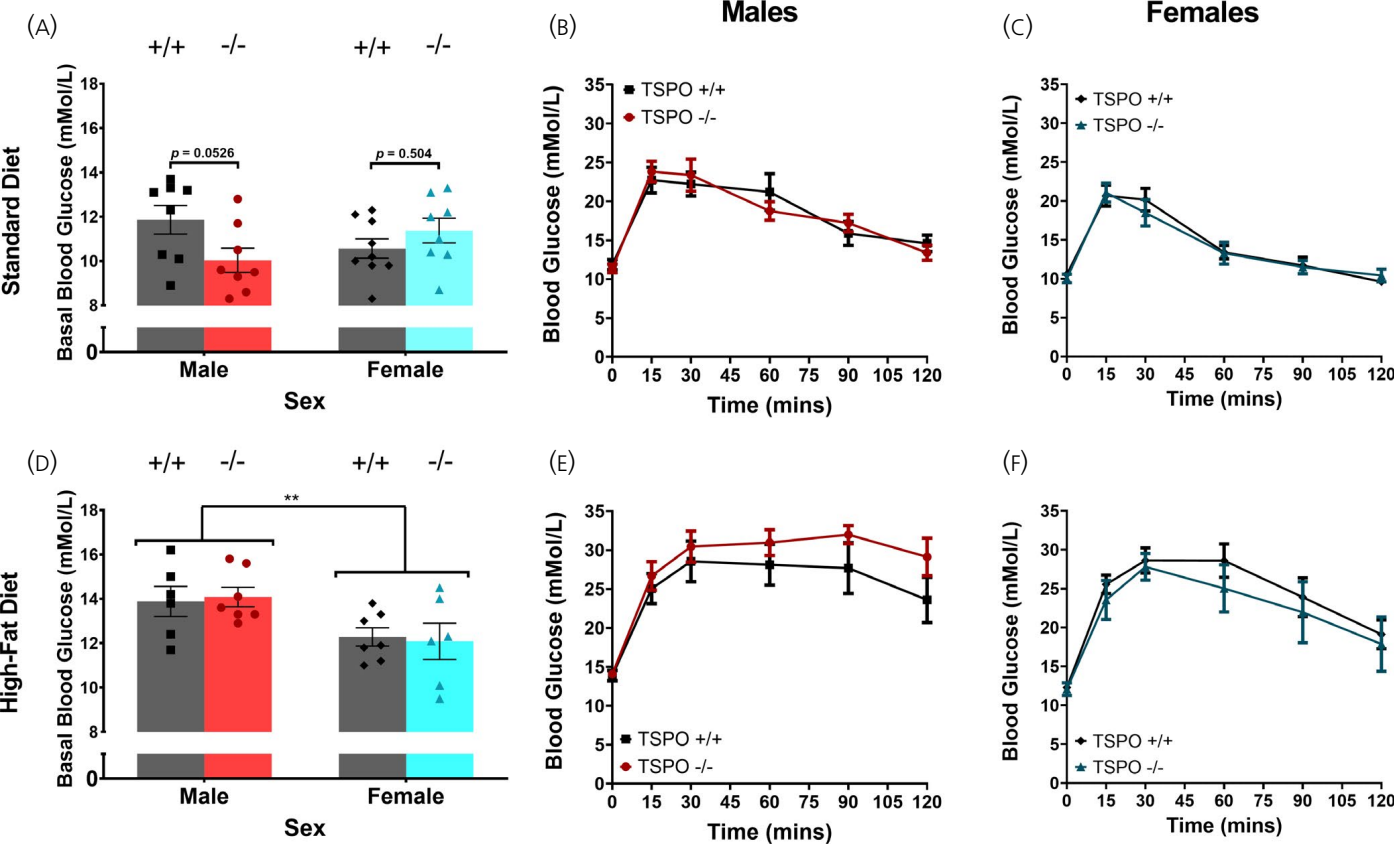
Germline deletion of TSPO had no impact on basal blood glucose or glucose clearance in a glucose tolerance test. TSPO^+/+^ and TSPO^−/−^ mice were administered an intra‐peritoneal bolus dose of 2 g kg^‐1^ glucose to assess blood glucose clearance at two time‐points in the study: 9‐10 weeks when fed standard chow diet (A‐C) and again after 5 weeks of a high‐fat diet (D‐F). Blood glucose levels were measured at 0 (basal blood glucose) and then 15, 30, 45, 60, 90 and 120 minutes after bolus glucose injection. There were no statistically significant effects of genotype alone on any of the parameters measured; however, in standard chow fed animals, there was a statistically significant interaction between sex and genotype (*F*_interaction 1,29_ = 5.839, *P* = 0.222) and Sidak's multiple comparisons test indicated that the effect in males was approaching statistical significance (*P* = 0.0526), whereas there was no effect in females (*P* = 0.5044). In the animals fed a HFD, female mice had a lower basal blood glucose level than male animals regardless of genotype (D) (*F*_sex 1,22_ = 9.40, **P = 0.0057; Two‐way ANOVA). Data are expressed as the mean ± SE, *n* = 6‐9 mice per group. Statistical analyses were performed using two‐way ANOVA (A and D) or a repeated two‐way ANOVA (B and C, D and E)

Intraperitoneal glucose tolerance tests were conducted at weeks 9‐10 of the study (Figure 1) to establish whether there was an effect of loss of TSPO on glucose homeostasis in animals fed standard chow. This was then repeated at week 17 of the study, following 5 weeks of exposure to the high‐fat diet to assess whether there was a genotype‐dependent effect of diet on glucose homeostasis. In standard chow fed animals, there was no statistically significant impact of sex or genotype on basal blood glucose levels (*F*_sex 1,29_ = 0.00145, *P* = 0.970; *F*_genotype 1,29_ = 0.870, *P* = 0.359, two‐way ANOVA) (Figure 5A). However, there was a statistically significant interaction between the two variables (*F*_interaction 1,29_ = 5.839, *P* = 0.222, two‐way ANOVA) (Figure 5A) and Sidak's multiple comparisons test indicated that the effect in males was approaching statistical significance (*P* = 0.0526), whereas there was no effect in females (*P* = 0.5044). Within sex, there was no effect of genotype on glucose clearance during the glucose tolerance test in the mice (*F*_genotype 1,14_ = 0.0037, *P* = 0.952 [males]; *F*_genotype 1,15_ = 0.064, *P* = 0.803 [females], both analysed using a repeated measures two‐way ANOVA) (Figure 5B).

After a HFD, there was an impact of sex but not genotype on basal blood glucose with female mice having a significantly lower level (*F*_sex 1,22_ = 9.40, *P* = 0.006; *F*_genotype 1,22_ = 2.011e^‐32^, *P* > 0.99; *F*_interaction 1,22_ = 0.119, *P* = 0.734, both analysed using a two‐way ANOVA) (Figure 5D). In the glucose tolerance test, there was no effect of genotype on glucose clearance in HFD fed mice of either sex (*F*_genotype 1,11_ = 1.290, *P* = 0.280 [males] [Figure 5E]; *F*_genotype 1,11_ = 0.429, *P* = 0.526 [females] [Figure 5F], both analysed using a repeated measures two‐way ANOVA).

When considering the relationships of all three independent variables (diet, sex, genotype) together using a three‐way ANOVA, there were statistically significant effects of diet alone and sex alone on both basal blood glucose and area under the curve (AUC) of glucose clearance in a glucose tolerance test. In the case of basal blood glucose, there was also a statistically significant interaction between diet and sex, with the female mice showing a comparatively lower increase in basal blood glucose after 5 weeks of a HFD than the male animals. These analyses are summarised in Table 1.

### 3.5 Germline global deletion of TSPO did not result in changes to liver nor peri‐gonadal adipose tissue composition in mice fed a HFD

**FIGURE 6:**
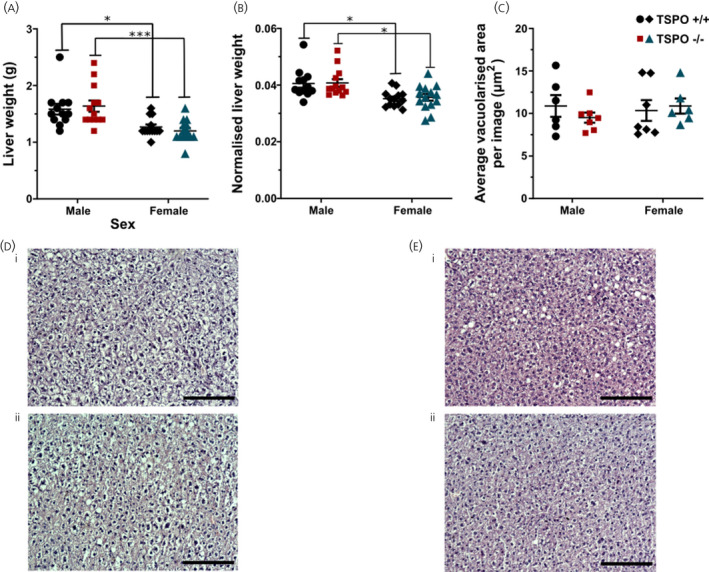
There were no differences in liver weight or mean vacuolarised area between high‐fat fed TSPO^+/+^ and TSPO^−/−^ mice. Liver weight (A), liver weight normalised to body weight (B) and mean vacuolarised area (C) (as a proxy for lipid accumulation) in male and female TSPO^+/+^ and TSPO^−/−^ mice. There were no statistically significant effects of genotype on any of the parameters measured. There was a statistically significant effect of sex on liver weight (A) (*F*_sex 1,48_ = 24.92, *P* < 0.0001) and liver weight normalised to body weight (B) (*F*_sex 1,48_ = 18.23, *P* < 0.0001). *P< 0.05, ***P<0.001. Statistical analysis was performed using two‐way ANOVA and Tukey's multiple comparisons test. Data are expressed as the mean ± SE, *n* = 11‐15 mice per group for the liver weight and *n* = 6 for the histology. Representative haematoxylin and eosin‐stained images of liver tissue structure from male TSPO^+/+^ (D‐I) and TSPO^−/−^ (D‐ii) mice, and female TSPO^+/+^ (E‐I) and TSPO^−/−^ (E‐ii) mice. Scale bars = 100 μm

Germline line global TSPO deletion did not affect the liver weight from the female or male mice (*F*_genotype 1,48_ = 0.009, *P* = 0.926, two‐way ANOVA) (Figure 6A), nor the liver weight as proportionate to body weight (*F*_genotype 1,48_ = 0.07, *P* = 0.8, two‐way ANOVA) (Figure 6B). As might be expected based on the differences in body weight, there was a statistically significant influence of sex on liver weight (*F*_sex 1,48_ = 24.92, *P* < 0.0001, two‐way ANOVA) (Figure 6A). Liver weight as a proportion of body weight was also influenced by sex (*F*_sex 1,48_ = 18.23, *P* < 0.0001, two‐way ANOVA) (Figure 6B), although there was no interaction between genotype and sex in either liver weight (*F*_interaction 1,48_ = 0.68, *P* = 0.416, two‐way ANOVA) (Figure 6A) or normalised liver weight (*F*_interaction 1,48_ = 0.02, *P* = 0.89, two‐way ANOVA) (Figure 6B). Analysis of mean vacuolarised space, as a proxy indicator of lipid droplet accumulation, in liver histology in male and female TSPO^+/+^ and TSPO^−/−^ mice indicated no effect of genotype (*F*_genotype 1,22_ = 0.17, *P* = 0.69, two‐way ANOVA) (Figure 6C) or sex (*F*_sex 1,22_ = 0.16, *P* = 0.69, two‐way ANOVA) (Figure 6C), nor an interaction between the two (*F*_interaction 1,22_ = 0.852, *P* = 0.366, two‐way ANOVA) (Figure 6C). Representative images of the liver histology are shown in Figure 6D for male TSPO^+/+^ and TSPO^−/−^ mice and in Figure 6E for female TSPO^+/+^ and TSPO^−/−^ mice.

**FIGURE 7:**
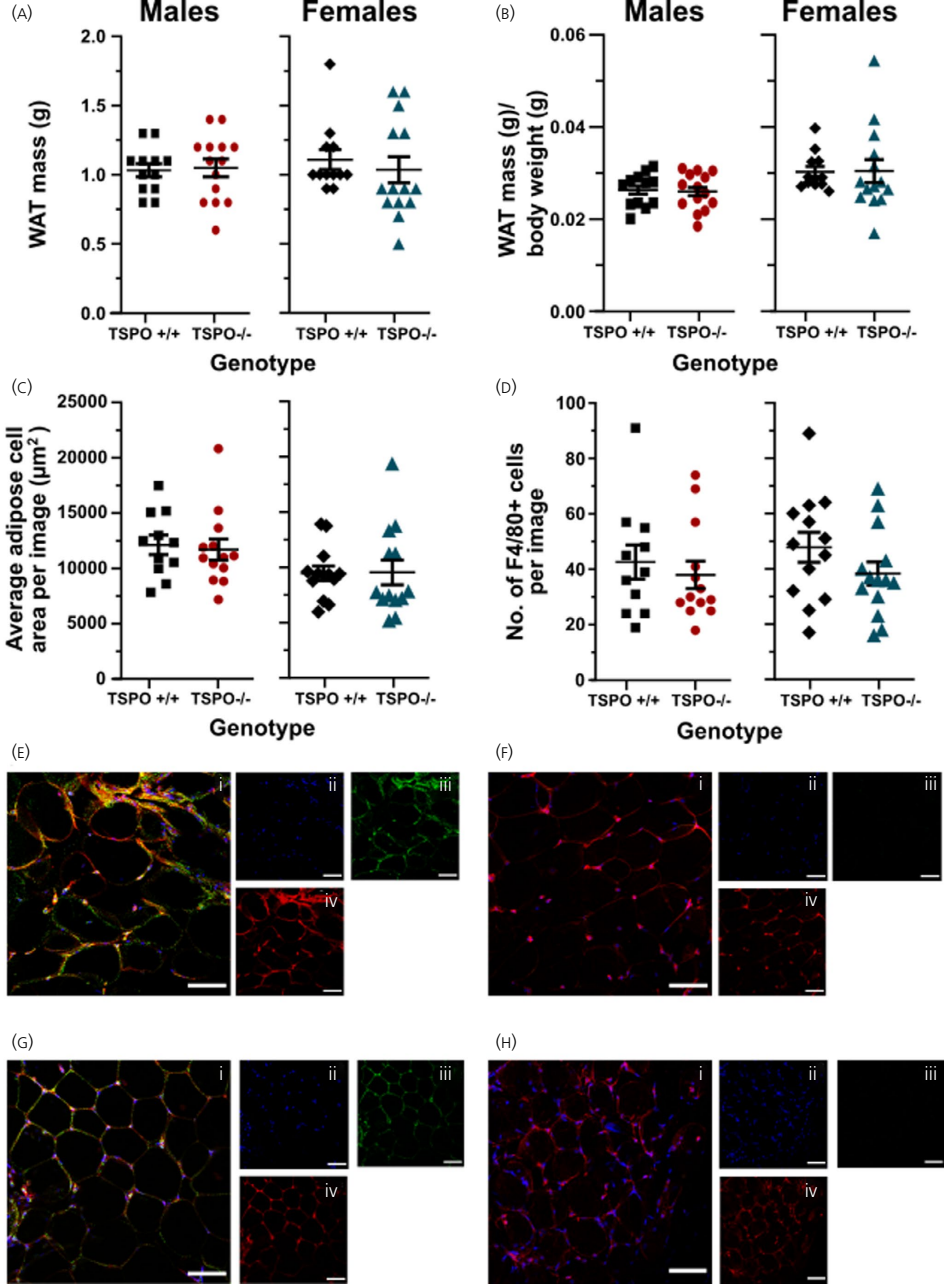
There were no differences in peri‐gonadal white adipose tissue (WAT) weight, mean adipocyte size or macrophage infiltration between high‐fat fed TSPO wild‐type and knockout mice. Peri‐gonadal WAT was dissected and weighed from male and female TSPO^+/+^ and TSPO^−/−^ high‐fat fed mice (A). WAT mass normalised to the corresponding body weight of the male and female mice (B). The mean adipocyte size between genotypes in either the male or female mice (C). Number of F4/80‐positive macrophages in WAT tissue (D). Statistical analysis was performed by either a Student’s or a Mann‐Whitney unpaired *t* test. There were no statistically significant effects of genotype on any of the parameters measured. Data are expressed as the mean ± standard error, *n* = 11‐15 mice per group. A representative 20× magnification image of WAT from a male (E‐I, merge) and a female (G‐I, merge) TSPO^+/+^ mouse ‐ depicting 4′,6‐diamidino‐2‐phenylindole‐positive nuclear staining (ii, blue), TSPO (iii, green) and F4/80 (iv, red) immunoreactivity. WAT immunohistochemistry from a male (F) and a female (H) TSPO^−/−^ mouse confirms the absence of TSPO immunoreactivity at 20× magnification (i‐iv). Scale bars = 100 μm

Immunohistochemistry was performed on the peri‐gonadal WAT dissected from the mice fed a HDF to quantify adipocyte size and visualise the expression of TSPO and the number of F4/80‐positive macrophages, a proxy marker of WAT inflammation. The absence of TSPO had no influence on the weight of WAT in the male (*P* = 0.842, unpaired *t* test) or female (*P* = 0.183, Mann‐Whitney *U* test) mouse cohorts, nor on the weight of peri‐gonadal WAT as a proportion of body weight (male cohort *P* = 0.824, unpaired *t* test; female cohort = 0.395, Mann‐Whitney *U* test) (Figure 7B). Absence of TSPO did not influence mean adipocyte size in either male (*P* = 0.749, unpaired *t* test) or female mice (*P* = 0.687, unpaired *t* test). There was no difference in the number of F4/80‐positive macrophages identified in WAT between TSPO^+/+^ and TSPO^−/−^ animals in both the males (*P* = 0.618, Mann‐Whitney *U* test) and females (*P* = 0.175, unpaired *t* test). As expected, TSPO immunoreactivity was expressed throughout TSPO^+/+^ tissue in both male (Figure 7Eiii) and female mice (Figure 7Giii) and absent in WAT from male (Figure 7Fiii) and female (Figure 7Hiii) TSPO^−/−^ mice. For WAT analyses, direct comparison between sexes was not used here as a result of the different anatomical localisation of the WAT depots examined: epididymal WAT was used for male animals and peri‐ovarian WAT was used for females.

## 4 DISCUSSION

In a genetically modified mouse line generated independently from the one presented here, previous research has examined the impact of global germline loss of TSPO in mice on normal growth.^9^, ^18^ Critically, for our hypothesis, the potential differences in the physiological response to alterations in energy availability in these animals were not explored. Based on published data indicating regulation of TSPO expression in rodents by obesity^13^ and a role for TSPO in modulation of mitochondrial function,^4^, ^5^, ^6^, ^7^, ^8^ we hypothesised that germline deletion of the *Tspo* gene in mice could impact the adaptive physiological response to alterations in energy availability.

Using the Tspo^tm1b(EUCOMM)Wtsi^ mouse line, we found that germline global loss of TSPO did not impact growth curves of male or female mice maintained on standard laboratory chow. This independently confirms the findings of Banati et al,^9^ and complements their study with measurements of weekly food intake. In the present study, animals with germline global loss of TSPO did not show any differences in their body weight or food intake in the negative (fast‐induced re‐feeding) or positive (5 weeks of a HFD) energy availability challenges employed. The male TSPO^−/−^ mice showed lower basal glucose levels on a standard diet, which was approaching statistical significance; however, we did not observe any genotype‐dependent differences in glucose clearance (AUC) in standard chow fed animals, nor basal blood glucose or glucose clearance (AUC) in animals after 5 weeks of a HFD. It is possible that a longer period of HFD feeding may have revealed an effect of germline global loss of TSPO on the parameters examined, particularly body weight gain, which potentially appeared to be moving towards divergence in female mice towards the end of our study. Indeed, three‐way ANOVA analysis of the body weight a data (comparing before and after HFD) revealed a statistically significant interaction between sex and genotype (sex × genotype), but not diet, sex and genotype (diet × sex × genotype). Unfortunately, limitations in our ethical approvals meant that the study could not be extended. As such, we cannot rule out an effect of germline global loss of TSPO on the response of mice to a more chronic HFD (> 5 weeks).

Five weeks of a HFD was sufficient to cause changes in glucose homeostasis in the mice that were studied, as indicated by an increased AUC in the glucose tolerance test, although this was seen in all animals regardless of genotype. Similarly, within sex, we saw no difference in gross weight or morphology of two key metabolic tissues (WAT and liver) between HFD fed mice of each genotype. Because we did not assess these parameters in animals maintained on standard chow, we cannot conclusively rule out genotype‐linked differences in the response of these organs to the HFD. Indeed, a study has reported that global germline TSPO deletion (*Amhr2*‐Cre‐mediated global TSPO knockout) results in neutral lipid accumulation in the gonads, adrenal glands and liver^34^; although there has been some debate regarding whether these animals truly represent a global TSPO deficient model.^35^ However, in the present study, no genotype‐dependent differences in WAT and liver were observed in the HFD‐fed animals, correlating with the results of the glucose tolerance tests.

Given the importance of mitochondrial function for survival, it is possible that developmental adaptations following germline deletion may functionally compensate for systemic loss of TSPO. Published studies have demonstrated an impact of tissue‐specific loss of TSPO expression on growth, energy and/or glucose homeostasis.^24^, ^36^ To modify TSPO expression exclusively in tanycytes in the mouse brain, Kim et al^24^ injected a viral vector containing cre‐recombinase into the third ventricle of 6‐week‐old TSPO *fl/fl* mice, resulting in knockdown of TSPO in these cells. Loss of TSPO in tanycytes in adult mice conferred relative protection from weight gain following a HFD, associated with reduced food intake and changes in energy expenditure, but, surprisingly, it did not improve glucose homeostasis in a glucose or insulin tolerance test.^24^ An independent study investigated the consequences of conditional TSPO knockout from predominantly steroidogenic tissues using the *Nr5a1*‐promotor in mice; this resulted in mice with alterations in growth and elevated blood glucose and lipids,^36^ which is postulated to be concomitant with alterations in adrenaline levels in this model.^19^ Pharmacological studies also support a potential role for TSPO in regulating glucose and/or energy homeostasis,^24^, ^37^ although these studies have used PK11195 which, at high doses, reportedly has off target effects.^38^ Further studies are needed with newer generation TSPO ligands with higher specificity and potency.^38^

To conclude, the observations made in the present study indicate, contrary to our hypothesis, that global germline TSPO deletion in mice does not affect body weight or food intake, including in response to the alterations in energy availability examined. One limitation of our study was the reliance on a germline deletion model, where developmental compensation/adaptations cannot be excluded. Future studies using adult inducible models and pharmacological approaches would be necessary to further test the hypothesis.

## AUTHOR CONTRIBUTIONS

**Nicole A Morrissey:** Data curation; Formal analysis; Investigation; Writing – original draft; Writing – review and editing. **Craig Beall:** Conceptualisation; Funding acquisition; Supervision; Writing – review and editing. **Kate LJ Ellacott:** Conceptualisation; Data curation; Funding acquisition; Investigation; Project administration; Supervision; Writing – original draft; Writing – review and editing.

## Data Availability

The data that support the findings of this study are available from the corresponding author upon reasonable request.
